# Tree Crown Mapping in Managed Woodlands (Parklands) of Semi-Arid West Africa Using WorldView-2 Imagery and Geographic Object Based Image Analysis

**DOI:** 10.3390/s141222643

**Published:** 2014-11-28

**Authors:** Martin Karlson, Heather Reese, Madelene Ostwald

**Affiliations:** 1 Centre for Climate Science and Policy Research, Department of Thematic Studies/Environmental Change, Linköping University, Linköping 58183, Sweden; E-Mail: madelene.ostwald@chalmers.se; 2 Section of Forest Remote Sensing, Department of Forest Resource Management, Swedish University of Agricultural Sciences, Umeå 901 83, Sweden; E-Mail: heather.reese@slu.se; 3 Centre for Environment and Sustainability, GMV, University of Gothenburg and Chalmers University of Technology, Göteborg 405 30, Sweden

**Keywords:** remote sensing, high spatial resolution, WorldView-2, tree crown mapping, tree crown delineation, geographic object based image analysis, woodland, agroforestry, parkland, Burkina Faso

## Abstract

Detailed information on tree cover structure is critical for research and monitoring programs targeting African woodlands, including agroforestry parklands. High spatial resolution satellite imagery represents a potentially effective alternative to field-based surveys, but requires the development of accurate methods to automate information extraction. This study presents a method for tree crown mapping based on Geographic Object Based Image Analysis (GEOBIA) that use spectral and geometric information to detect and delineate individual tree crowns and crown clusters. The method was implemented on a WorldView-2 image acquired over the parklands of Saponé, Burkina Faso, and rigorously evaluated against field reference data. The overall detection rate was 85.4% for individual tree crowns and crown clusters, with lower accuracies in areas with high tree density and dense understory vegetation. The overall delineation error (expressed as the difference between area of delineated object and crown area measured in the field) was 45.6% for individual tree crowns and 61.5% for crown clusters. Delineation accuracies were higher for medium (35–100 m^2^) and large (≥100 m^2^) trees compared to small (<35 m^2^) trees. The results indicate potential of GEOBIA and WorldView-2 imagery for tree crown mapping in parkland landscapes and similar woodland areas.

## 1. Introduction

**Figure 1.**
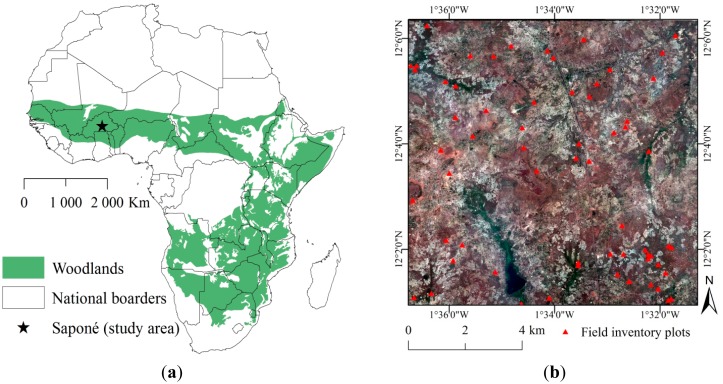
(**a**) Distribution of woodlands in Africa and location of study site (source: modified from White [2]); (**b**) WorldView-2 image of the study area and location of field plots.

With a spatial extent of close to 9 million km^2^ [1,2], woodland is the most extensive vegetation type in Africa (Figure 1). Since approximately 60% of the woodland area in Africa is under cultivation [3], this vegetation type represents the subsistence base for the majority of the rapidly growing population [4,5]. In semi-arid West Africa, small scale shifting cultivation in traditional agroforestry systems constitutes the main agricultural practice [6,7]. Such modified and managed woodland landscapes are locally referred to as parklands [6,8]. Discontinuous tree cover is a critical component of the parklands [6,9] as it controls a number of key ecosystem service functions (e.g., microclimate and soil properties, including soil water dynamics and carbon sequestration) and provides important subsistence resources (e.g., food, fodder, medicine and fire wood). Recently, concerns have been raised that changing climatic conditions are causing tree cover degradation (with diminishing tree densities and altered species composition) in this region [10–12]. A strong increase in population, and demand for agricultural land, tree products and grazing land also exert pressure on the tree cover and constrain tree regeneration [13–15]. At present, a general shortage of tree cover data impairs the preconditions for scientifically sound research and monitoring needed for informed management decisions in parkland and other African woodland areas [1,16]. Since detailed field based tree inventories are highly labor- and cost-intensive, and problematic to conduct on broad scales, alternative approaches to tree cover data collection are needed.

### 1.1. Mapping Tree Cover with High Spatial Resolution Satellite Imagery

Remote sensing has become a key tool for broad scale analysis of forest and woodland ecosystems. The improved level of detail from high spatial resolution (HSR; ≤4 m) satellite imagery has expanded the possibilities to map structural and floristic attributes of forest stands, as well as individual trees [17–19]. Accurate detection and delineation of individual tree crowns (ITC) in HSR imagery is a critical step for remote sensing based tree inventories because it generates the basic unit of measurement upon which other structural and demographic attributes are based [20], including crown area (CA; m^2^), tree density (TD; trees/ha) and tree canopy cover (%). Mapping the distribution of ITC across large areas holds potential for a wide range of research and management applications. For example, tree crown size (area, radius or diameter) has been used to model structural attributes, such as basal area, diameter at breast height (DBH) and above ground biomass through allometric relationships [21–23]. Tree crown delineation is also a key step in remote sensing based tree species discrimination [24], and for spectral assessments of tree cover condition, for example water stress [25], because its accuracy controls the quality of the extracted reflectance spectra [20,26]. It is noteworthy that numerous studies have reported significantly higher accuracies when tree species classification is performed on the crown level as compared to pixel-level approaches, e.g., [26–29].

For detailed tree cover mapping, HSR satellite imagery constitute an economically and practically feasible alternative to airborne systems, such as hyper-spectral imagers and Light Detection and Ranging (LiDAR), as access and resources for these are often limited in Africa [28,30]. A range of commercially operated satellite systems (e.g., Quickbird, OrbView, WorldView and GeoEye) now provide, on request, global coverage of panchromatic and multispectral imagery at high spatial resolution, enabling analysis on the ITC scale [31–34]. While such HSR imagery has been successfully used for inventories of high latitude conifer forests [17–19,34], its applicability in tropical ecosystems has received limited scientific attention [35–37].

### 1.2. Techniques to Detect and Delineate Tree Crowns in HSR Imagery

Many methods to automate tree crown detection and delineation in HSR imagery have been developed over the years. Tree crown detection has commonly used techniques based on local maxima filtering [38], adaptive binarization [39] and template matching [40]. Tree crown delineation has been realized through different types of segmentation algorithms, including region-growing [41], valley-following [42] and watershed segmentation [43]. Common for all these approaches is that they use the same basic assumption for pattern recognition: that the geometric centers of illuminated crowns (tree tops) appear brighter than crown edges in HSR imagery (see e.g., [34,41,44]). Research on automated tree crown detection and delineation has primarily been conducted in North America and Northern Europe, targeting applications in managed coniferous forests often characterized by a relatively homogeneous structure with uniform tree sizes, age classes, spacing, species compositions, crown shapes, and a contrasting field layer. Such a tree cover structure translates to relatively consistent radiometric patterns in HSR imagery, facilitating the implementation of automated methods [45]. Consequently, the majority of automated tree crown mapping methods are less applicable in areas where the tree cover is structurally complex and where deciduous tree species dominate [20,44]. Such complex tree cover conditions are found across large parts of the African woodlands, including the parkland-landscapes of West Africa.

Five main factors introduce complexity to the spectral response of parkland tree cover that complicate automated crown mapping in HSR imagery [41,44]: (1) trees often have similar leaf area index (LAI) to that of shrubs and herbaceous vegetation, making separation based on spectral information difficult [46,47]; (2) the field layer is often heterogeneous in terms of soil color and vegetation density due to local topographic and edaphic conditions and land use; (3) local environmental conditions and land use also introduce large variations in the tree cover structure, including highly variable trees sizes, densities and spacing; (4) deciduous tree species generally support a structurally complex crown that causes high within-crown brightness variations in HSR imagery due to the heterogeneous reflectance of branches and related shadows; and, (5) the spectral variation within and between tree species may be high due to differences in leaf reflectance properties (e.g., foliar biochemistry, moisture content and structure) resulting from local environmental conditions, phenological variations and evolutionary adaptation traits of semi-arid vegetation [44].

### 1.3. Moving from Pixel-Level to Object-Level Information

The previously mentioned factors indicate that the spectral content of individual pixels is not sufficiently consistent for accurately characterizing parkland tree cover in HSR imagery. To overcome these problems, spatial information in HSR imagery, including geometry (e.g., shape and size), texture and context, can be exploited when designing algorithms for tree crown delineation [41,43,48]. Geographic object based image analysis (GEOBIA) has been proposed as a suitable approach for a wide variety of complex feature recognition problems in remote sensing [49], including tree crown delineation [21,44,49]. Within GEOBIA, regions of spectrally similar and spatially connected pixels (objects) form the units for data processing, instead of individual pixels. The image objects that are generated by image segmentation techniques contain geometric, textural, contextual, as well as spectral information [50,51]. From a theoretical perspective, GEOBIA appears to be a well-suited approach for automated tree crown delineation in parkland landscapes for several reasons. In particular, GEOBIA facilitates feature recognition at multiple spatial scales [52]. This means that crown delineation algorithms can be adapted to handle both groups of trees and individual trees despite large size variations. GEOBIA is also suitable to counter the H-resolution problem [51,53] of high within-class spectral variability inherent to HSR imagery in general, and areas of heterogeneous tree cover in particular. GEOBIA can thus improve mapping accuracy and reduce effects of “salt-and-pepper” speckle, which is a common problem in pixel based analysis.

Previous research suggests that the information within objects is highly useful for separating different vegetation types with similar spectral signatures [54] and for detection and delineation of tree crowns in complex environments [44,49]. While the GEOBIA approach has shown promise in African woodland landscapes for satellite based mapping of aggregated tree cover variables, such as tree canopy cover [21,55], its use for the delineation of ITC has not been extensively evaluated in the tropics [34]. An exception to this was provided by Bunting and Lucas who applied a rule-based GEOBIA approach to delineate ITC and crown clusters in an Australian woodland area (Queensland) using hyper-spectral imagery from the Compact Airborne Spectrographic Imager (44).

### 1.4. Research Objectives

The research presented in this article has two objectives: (1) develop a tree crown delineation method for application with HSR satellite imagery in African parkland landscapes using GEOBIA; and (2) conduct a detailed accuracy assessment of the tree crown delineation method using field reference data.

The article addresses the following question: how effective is the combination of GEOBIA and HSR satellite imagery for automating tree crown delineation in a parkland landscape? This is assessed in terms of: (i) the degree of agreement between reference trees, including their crown area, and tree crowns delineated by the automated method; and (ii) the overall correspondence between plot level tree canopy cover (*i.e.*, aggregated crown area) derived from the reference trees and the delineated trees. We will also analyze the types of errors (e.g., omission and commission errors) and the potential causes (e.g., crown dimension, tree species and land use).

## 2. Study Area and Data

### 2.1. Geographical Background

The 10 km × 10 km study site is located on a low relief plain (293–363 m above sea level) 35 km south of Ouagadougou within the rural commune of Saponé (Bazega province; 12°04′ 48″ N, 1°34′ 00″ W). The local climate is bimodal with a rainy season taking place between April and October, followed by a long dry spell. Annual mean rainfall is approximately 790 mm· year^−1^ (1952–2010) and the annual levels range between 570 and 1180 mm· year^−1^, with about 80% of the rainfall falling during June through September (Direction de la Météorologie du Burkina Faso). The mean annual temperature (1952–2008) is 28 °C and the mean annual potential evapotranspiration is 1900 mm·year^−1^ (1974–2003), which means that the climate is semi-arid according to the Köppen-Geiger climate classification system [56]. The soils of the area are sandy loamy Regoslos with low nutrient content [57]. Biogeographically, Saponé is located in the Sudano-Sahelian ecotone [58].

**Figure 2.**
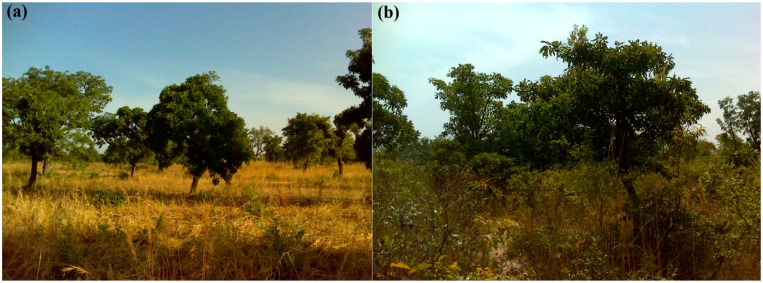
Picture showing examples of (**a**) active field (**b**) and fallow dominated by *Vitellaria paradoxa*.

The landscape of the study site consists of a mosaic of settlements, active and fallowed rain-fed farm land (*i.e.*, parklands), scattered tree plantations and patches of dense woodland (e.g., riparian formations). The parklands are dominated by the traditional agroforestry tree species *Vitellaria paradoxa*, *Parkia biglobosa* and *Lannea microcarpa*, and used for cultivation of staple crops, such as millet, sorghum (red and white) and maize, together with legumes (e.g., cow pea and peanut). Other well known agroforestry tree species found in the area include *Adansonia digitata, Faidherbia albida* and *Bombax costatum*,*Vitellaria paradoxa* and *Parkia biglobosa* are generally considered deciduous but are rarely leafless because of a progressive replacement of the leaves [6]. Lannea species *(microcarpa* and *acida), Adansonia digitata* and *Bombax costatum*, on the other hand, shed leaves in the dry season [59]. *Faidherbia albida* has a reverse phenology: it foliates during the start of the dry season and sheds leaves early in the wet season. The fields are regularly fallowed for 3–5 years. In comparison to active fields, the fallows are characterized by a higher tree density and species diversity, and a denser understory composed of annual grasses, shrubs and tree re-growth (Figure 2).

### 2.2. Satellite Data and Preprocessing

A WorldView-2 image was acquired under cloudless conditions on 21 October 2012 (processing level: Ortho Ready Standard, scan direction: forward). This date represents the early dry season when most of the tree species have fully developed foliage. The WorldView-2 satellite provides data in one panchromatic band and 8 spectral bands (Table 1.). The ground sampling distance at nadir is 0.5 m for the panchromatic band and 2 m for the multispectral bands.

Digital numbers of the nine bands were converted to top-of-atmosphere radiance using the embedded absolute radiometric calibration factors and effective bandwidths, according to the specifications provided in [60]. The Hyperspherical Color Space (HCS) algorithm [61] and a 7 × 7 pixel smoothing filter was used to fuse the multispectral bands with the panchromatic band, resulting in an 8-band pan-sharpened image with a pixel size of 0.25 m^2^. The HCS algorithm was developed specifically for use with WorldView-2 data and implemented in ERDAS Imagine 2013 software. The pan-sharpened image was rectified to UTM 30N using 35 ground control points collected in the field and third order polynomials, with a root mean square error (RMSE) of less than one pixel. The satellite data served two purposes in this study: (i) to support the establishment of field inventory plots; and (ii) to map tree crowns using GEOBIA.

### 2.3. Field Reference Data

A field inventory was conducted in November 2012 where a total of 64 square inventory plots (50 m × 50 m) were collected. Field data collection followed a stratified random sampling procedure in order to ensure coverage of the different tree densities in the study area. The stratification was achieved by partitioning the study areas into four classes of tree canopy cover (CC; Table 2) using the Normalized Difference Vegetation Index (NDVI) data [62] derived from the WorldView-2 image and a moving window approach [63]. Samples were not taken from the first strata (0% CC) since these were areas without trees (validated in the field campaign). Even though the stratification was based on a rather crude estimation of tree canopy cover, it captured the characteristics of the study site well. An approximately even number of plots per strata with CC > 0 were randomly distributed throughout the parklands of the study area.

Plot centers were located in the field using a Global Positioning System (GPS; Garmin Oregon 550, Garmin, Olathe, KS, USA) receiver and printed true-color composite maps of the study area. For each plot the current land use was noted. Trees with a DBH ≥ 5 cm were surveyed for the variables listed in Table 3. Ground projected crown area (CA) was calculated by assuming an elliptical crown shape using Equation (1) [64]. In order to account for the positional uncertainties related to the GPS recordings, each point was manually related to a tree in the image using information on crown dimensions, height and species as guidance.

(1) $$\text{CA}=\;\pi \;\left(\frac{\text{D}1}{2}\times \frac{\text{D}2}{2}\right)$$

A total of 36 tree species were identified during the field inventory. The four most abundant tree species (*Vitellaria paradoxa n* = 207; *Lannea microcarpa n* = 61, *Mangifera indica n* = 52 and *Parkia biglobosa n* = 29) represented 70% of the sample. The tree sample (*n* = 497) had a mean CA of 38 m^2^ (min = 1 m^2^; max = 606 m^2^, s.d. = 56.9), a mean height of 6.9 m (min: 1.5 m; max: 25 m, s.d. = 3.5) and mean DBH of 28 cm (min = 3.5 cm; max = 143 cm; s.d. = 22.4). *Vitellaria paradoxa* and *Lannea microcarpa* cover the whole size range in the sample and are characterized by compact crowns [59]. *Mangifera indica* and *Parkia biglobosa* trees are generally large (e.g., > mean CA), where the former support a compact crown and the latter are characterized by expansive branching. Many of the species in the parkland system is pruned for fire fuel and browse [6] which introduces further complexity in the shape of the tree crowns. Eighteen of the 50 m × 50 m field plots, chosen to capture the environmental complexity of the landscape, were used as reference data to guide the delineation of the tree crowns. The remaining 47 plots were used as an independent validation dataset (see Section 3.1.5).

## 3. Methods

### 3.1. Automated Delineation of Tree Crowns

**Figure 3.**
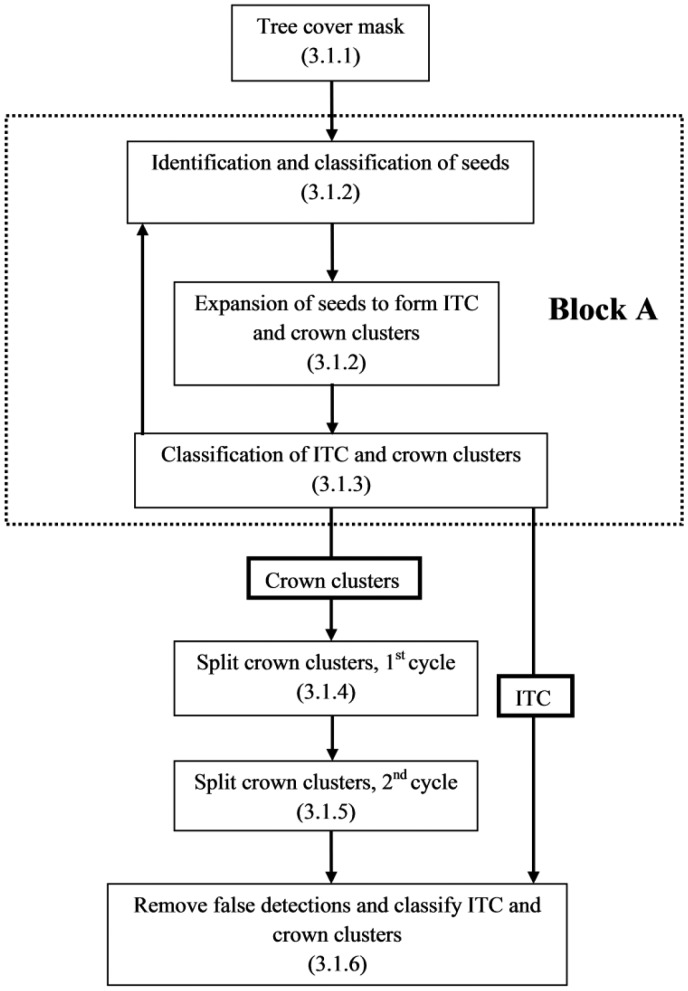
Flowchart of the GEOBIA processing steps and their sections in the paper. Block A (inside the dotted line) is repeated to split crown clusters (Section 3.1.4).

The GEOBIA approach for tree crown delineation was implemented in eCognition^®^ Developer 8.8 (Trimble) software. The algorithms applied for segmentation and the object features used for classification are briefly described in the following section, and further described in the application reference book [65]. Suitable features for object based classification were identified using the exploratory tools of eCognition. The GEOBIA approach consists of six main steps that are applied and iterated according to the flowchart shown in Figure 3. Inspiration to this approach was derived from the work of Bunting and Lucas [44].

#### 3.1.1. Tree Cover Mask

The first step aims to mask out non-tree cover, including water, bare soil, man-made constructions, crops and understory vegetation (*i.e.*, grass and shrubs). A range of spectral vegetation indices, such as the NDVI, the Enhanced Vegetation Index [66] and the Forest Discrimination Index [44] were assessed for their suitability to distinguish between tree cover and non-tree cover pixels using the 18 training areas as reference data. However, none were found to provide acceptable results; conservative thresholds resulted in large areas of understory vegetation being included in the tree cover mask, whereas restrictive thresholds excluded a high proportion of trees, in particular species characterized by a low LAI. We therefore propose to base the tree cover extraction on spatial and spectral information derived from image objects that capture tree cover characteristics better than vegetation indices applied on the pixel level. Image objects were first generated by applying a threshold to the NDVI and subsequently classified according to the criteria of Table 4. This procedure is iterative; it starts at a low NDVI threshold (*min_thresh_*; 0.05) with stepwise increments (0.05) until a threshold (*max_thresh_*: 0.2) is reached. *Min_thresh_* and *max_thresh_* are determined by referring to the training areas. After each NDVI increment the pixels with values < *min_thresh_* are assigned to *background* whereas the remaining spatially connected pixels are merged to form *potential tree mask* objects. Only objects having an area < 1500 m^2^ are considered for classification in order to prevent inclusion of large areas with a high proportion of background classes. The roundness and elliptical fit features were used to identify objects approximating a circular shape likely to represent a single crown or a small group of crowns. The roundness of an object is calculated as the difference between the enclosing and the enclosed ellipse, where a value of 0 represents a perfect ellipse and values > 0 indicate a more irregular shape [65]. The elliptic fit feature superimposes an equaled area ellipse on the objects and the area of the object outside of the ellipse is compared to the area inside the ellipse, with values ranging between 0 and 1 (*i.e.*, perfect ellipse). Since tree crown reflectance is influenced by both photosynthetic and non-photosynthetic material (*i.e.*, leaves and branches), these objects often have higher spectral variation than for other vegetation types, in particular grass [49]. Thus, we used the object level standard deviation (s.d.) of NIR reflectance as an indicator of spectral variation to distinguish tree cover objects.

At this stage a significant amount of field layer vegetation was included in the tree mask and further refinement was therefore needed. First, multi-resolution segmentation [50] was performed on relatively large objects within the tree mask, with the size constraint (set to > 700 m^2^) and the scale parameter (set to 20) set sufficiently high to prevent over-segmentation of ITC. The object size threshold was set with reference to the largest trees in the training dataset, whereas the scale parameter was determined through a trial and error approach. The scale parameter (unit less) controls the degree of spectral variation within objects and therefore their resultant size. Over-segmentation of crowns results in spectrally homogeneous objects and thus dilutes the features (spectral variation) upon which the tree cover classification is based. The resulting objects were assessed for their spectral properties where a low NIR s.d. characterized background objects with a homogeneous canopy (*i.e.*, low spectral variability) such as patches of grass and crops. A final refinement of the tree cover mask was then performed to remove remaining field layer vegetation, in particular shrubs and tree regrowth. This was achieved by performing an ISODATA [67] unsupervised classification on the WorldView-2 image using all eight bands within the tree cover mask, with the number of potential clusters set sufficiently high (set to 50) to limit class mixing. Clusters representing understory vegetation were identified manually and these pixels were subsequently removed from the tree cover mask.

#### 3.1.2. Object Maxima Identification, Classification and Expansion

**Figure 4.**
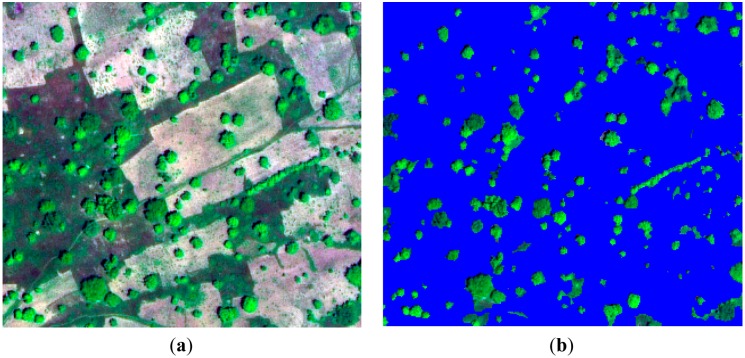
(**a**) True color WorldView-2 image of active fields (bright background) and fallows (dark background); (**b**) tree cover mask and non-tree cover areas (blue).

Following tree cover extraction, all adjoining tree mask objects (Figure 4) were merged. At this point small background objects (<50 m^2^) enclosed by tree mask objects were reintegrated. Such small background objects generally represented hollow, shadowed and directly sun-lit sections within large tree crowns. Within each of the individual tree mask objects a radiometric maximum (assumed to represent the tree top) was identified. Radiometric maxima were identified using NDVI and served as the starting points (*i.e.*, seeds) for region-growing segmentation [41,44]. We located seeds using 2 m pixels (*i.e.*, WorldView-2 multispectral) instead of the 0.5 m pan-sharpened pixels for two reasons. Firstly, seeds based on 2 m pixels were more consistently located towards the geometric centre of tree crowns compared to the 0.5 m pixel where within-crown variation made seed location more inconsistent (e.g., located on crown edges). Secondly, 2 m pixels provided a more representative characterization of the crown on which the seed was located. The mean NDVI of the seeds was used to classify the seeds into three tree species type categories (Table 5). This process guided the parameterization of the region-growing algorithm to account for tree species-type dependent variations in crown characteristics (e.g., LAI and leaf reflectance properties). A low NDVI threshold (<0.1), determined from the training dataset, was set in order to minimize incorrect seed identification occurring from sparse field layer vegetation or bare ground.

A region-growing process was then initiated where each seed expanded into adjacent 0.5 m pixels. The growing process was constrained by two spectral thresholds (Table 5) based on the difference in NDVI and NIR between the original seed and the adjacent candidate pixels: a sharp decrease in the NDVI and NIR values indicated crown edges. The NDVI is relatively insensitive to within-crown brightness variations and therefore suitable for detecting true crown edges while minimizing over-segmentation [49]. On the other hand, previous research suggests that NIR reflectance is particularly suitable to locate the limit between crown edges and shadows [41], thereby making it a good complement to the NDVI threshold. The spectral thresholds were optimized for tree species types as determined from the training areas. For example, trees with low LAI (*i.e.*, low seed NDVI) contrasted less with the background; hence a lower threshold was required to prevent the region-growing algorithm to include non-tree pixels. After termination of the initial growing process a new seed was located and expanded according to the procedure described above. This process was iterated until each tree mask object was contained by potential single tree crown (*i.e.*, “ITC”) or multiple crown objects (*i.e.*, crown cluster).

#### 3.1.3. Classification of Crowns and Crown Clusters

The objects generated by the region-growing segmentation can either represent an ITC, or a crown cluster that needs to be further segmented. The stage presented in this section separates ITC from crown clusters by use of geometric object features. ITC and crown clusters were classified based on features that characterize object shape, with thresholds determined from the training data. We used the ratio of length to width to identify asymmetric or elongated objects (threshold > 1.7), which were likely to represent crown clusters. The roundness feature was used to identify highly irregularly-shaped objects (threshold > 0.6). Object area was used to limit the possible extent of the delineated tree crowns (threshold > 700 m^2^). Objects considered to represent ITC were temporarily removed from the data (see Figure 3), allowing those objects identified as crown clusters to be further segmented into ITC (procedures outlined in following sections).

#### 3.1.4. Splitting Crown Clusters, 1st Cycle

In order to extract ITC from objects classified as crown clusters, the steps (Section 3.1.2; Section 3.1.3) of Block A (Figure 3) were iterated in two cycles where the spectral thresholds (Table 5) used for the region-growing algorithm were decreased stepwise (via multiplication with a factor of 0.75). By decreasing the spectral thresholds, more subtle crown edges could be detected, facilitating crown delineation in areas where contrast with the adjacent field layer was low. Two cycles of crown cluster splitting were used, based on a compromise between processing time and enabling crown delineation for varying field layer conditions.

#### 3.1.5. Splitting Crown Clusters, 2nd Cycle

The objects still classified as crown clusters after the two cycles (3.1.4) were considered problematic to split based on spectral thresholds. Thus, we applied morphological watershed segmentation that splits objects based on shape characteristics instead of spectral gradients [68]. More specifically, for each pixel contained within the individual crown cluster object, the inverted distance to the closest object border is calculated. The resulting local minima serve as seeds in a region-growing that terminates when the borders of the new objects meet. The watershed segmentation is most suited to delineate ITC of elongated and sprawling crown clusters which conform to the assumptions of the algorithm [49]. It may, however, cause over-segmentation of compact crown clusters resulting in inaccurate crown delineation.

#### 3.1.6. Removal of False Detections and Classification of Crowns and Crown Clusters

In the final step of the process, the delineated objects undergo two classification procedures which separate false detections, ITC and crown clusters. Firstly, false detections are identified and removed using NIR and RE s.d. thresholds (Table 4). Secondly, the objects derived from the crown cluster splitting procedures (3.1.4 and 3.1.5) are evaluated according to the geometric features of Table 4. Adjacent objects classified as crown clusters are subsequently merged to correct for over-segmentation of compact objects. We considered the splitting of such crown clusters to be beyond the capabilities of the WorldView-2 data and the proposed GEOBIA approach.

#### 3.1.7. Accuracy Assessment

In order to assess the reliability of the method we compared the delineated tree crowns with an independent reference dataset representing individual tree positions with associated attributes (e.g., species, DBH and CA). Initial trials revealed that automated matching of field trees and delineated crown objects was difficult due to the complex and multilayered tree cover structure. Thus, an accuracy assessment was performed where field trees and delineated crown objects were associated manually. The assessment focuses on two aspects of accuracy: tree detection accuracy and crown delineation accuracy.

To assess tree detection accuracy (Table 6), we recorded the number of field trees that were clearly associated with a single crown object presenting a one to one relationship (individual tree detection; ITD), the errors of omission (EO) and the errors of commission (EC). The overall detection accuracy was based on Poulion *et al.*'s (2002) accuracy index [69], defined as:

(2) Accuracy Index (%) = (n − (O + C))/n  × 100

where O and C represent the numbers of omission and commission errors, respectively, and n represents the total number of field trees in the accuracy assessment dataset. We also recorded the cases where multiple field trees were contained by a single crown object, including the number of trees in the crown cluster (crown cluster detection; CCD).

For assessing the crown delineation accuracy, the CA of field trees was compared to the CA of the delineated crown objects, for both ITC and crown clusters. The CA of the individual field trees associated with crown clusters (CCD) was aggregated to enable quantitative comparison to the delineated objects. In order to assess the delineation, three measures of accuracy were used: (1) the correlation coefficient (Spearman's Rho; *r_s_*) between field CA and delineated CA; (2) the mean absolute error (MAE) between field CA and delineated CA [70]; (3) the mean relative error (MRE), defined here as the averaged ratio of the absolute error to field CA; and, (4) the mean bias error (MBE), defined here as the difference between the mean delineated CA and the mean field CA, which indicates the degree of over- or under-estimation. The statistical analyses were performed using the Statistical Package for the Social Sciences (SPSS) 21 software.

We assessed the delineation accuracy for ITC, crown clusters and tree canopy cover. For the ITC, three crown size classes were assessed separately: small trees (CA < 35 m^2^), medium trees (CA 35–100 m^2^) and large trees (≥100 m^2^). Separate assessments stratified by land use (*i.e.*, active field and fallow) were also performed. To assess the accuracy of tree canopy cover estimation, plot level aggregates of field tree CA were compared to the CA of all delineated crown objects, including the errors of commission.

## 4. Results and Discussion

### 4.1. Detection Accuracy

When detection accuracy is defined as a 1:1 correspondence between field trees and delineated objects (ITD), the results of this study are modest: 48.4% of the trees > 5 cm and 53.8% of trees > 10 cm DBH were detected as ITC (Table 7). Detection accuracy of ITC appears to be affected by the vegetation conditions in the fallow plots, as reflected by the lower detection rates. This finding is in line with research conducted in other woodland areas, which suggests that detection rates normally decline with increasing tree density [41,44]. When the definition of accuracy is relaxed to also include crown clusters as correct detections, the results improved to 85.7% of the trees > 5 cm DBH being correctly detected. Allowing crown clusters as a mapping unit is a reasonable approach in this situation considering the patchiness of the tree cover structure [41,44] and the resolving power of the WorldView-2 imagery.

Our results compare well to similar studies that have applied GEOBIA for tree crown mapping and have considered crown clusters as correct detection. For example, Ardila *et al.* reported a detection rate between 70%–80% using Quickbird imagery to map urban tree cover in the Netherlands [48], and Bunting and Lucas reported a detection rate of 71% using Compact Airborne Spectrographic Imager (CASI) imagery in Australian woodlands [44]. Also using CASI imagery, but applying the valley-following approach [42], Leckie *et al.* achieved a detection rate between 50%–60% for an old growth conifer area in Canada [20]. Higher accuracies have been reported in less complex tree cover conditions, for example by Pouliot *et al.*, who achieved a detection rate of 91% for a spruce plantation using a modified local maxima approach and multispectral aerial imagery [69].

**Figure 5.**
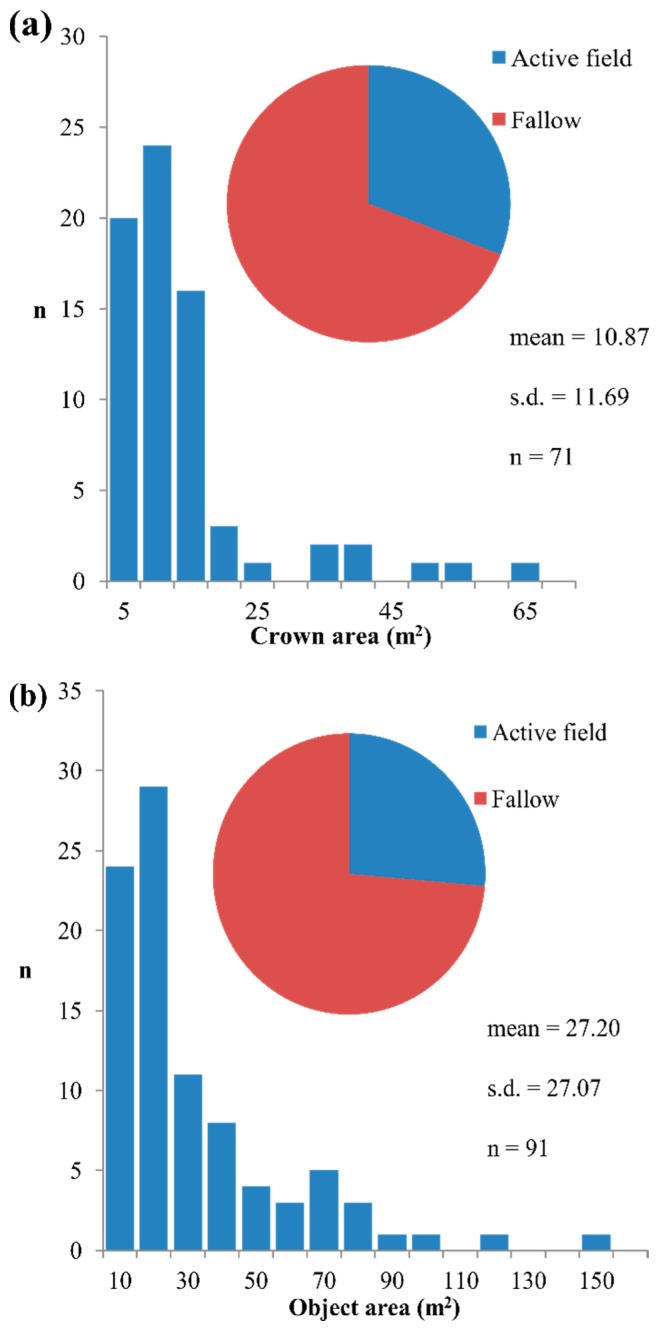
Diagrams presenting statistics for (**a**) omission errors and (**b**) commission errors. The bar-charts show the errors distributed in size classes, whereas the pie-charts show the distribution of errors in the two land-use categories (active field and fallow).

The total proportion of omission errors (total = 14.3%) is acceptable in both active field (11.6%) and fallow plots (12.3%), with omissions attributed mainly to missing small trees with CA < 15 m^2^ (Figure 5a), which corresponds to an area less than four multispectral WorldView-2 pixels (2 m). No consistent relationship was found between tree species and the errors of omission which suggests that the WorldView-2 imagery was acquired during a stable phenological stage (early dry season) when the deciduous woodland tree species have favorable canopy conditions for remote sensing analysis. These results also suggest that the method used to create the tree cover mask (Section 3.1.1) enables an acceptable separation between tree cover and field layer objects (e.g., grass, shrub and tree re-growth) that are spectrally similar on the pixel level and therefore problematic to separate using vegetation indices or pixel based classification. In particular the geometric information facilitated the inclusion of small trees and tree species with low LAI (*i.e.*, partly senescent crowns whose reflectance is highly influenced by the field layer), which are easily misclassified with purely spectral approaches.

The total proportion of commission error is 18.3% (Figure 5b), and occurs most for objects with an area below the reference sample mean (*i.e.*, <38 m^2^). The error rate is considerably lower in active fields (12.3%) as compared to fallows (21.7%) where small tree re-growth (<5 cm DBH) is more abundant. The relatively high error rate is expected due to the similarities in the spectral response between tree crowns and tree re-growth, especially when the latter is arranged in crown clusters that exceed the extent of the 2 m multispectral WorldView-2 pixels (*i.e.*, >4 m^2^). Since the field reference dataset was restricted to trees with DBH > 5 cm to ensure a reasonable workload, the interpretation of the commission error rate is not straight forward and could to some extent conceal detections of small trees. However, high commission error is a well-known limitation and therefore a central issue for research on tree crown detection in HSR imagery [34]. Unfortunately, quantifications of commission errors are not reported as frequently in the literature as detection accuracy (including error of omission), which reduces the possibilities for inter-study comparisons. The commission error rate in the present study (18.3%) is lower than that reported for comparable tree cover conditions, for example 26% for urban tree cover in the Netherlands [48] and 25% in Canadian old growth forest [20]. Lower rates of commission errors (3.0%–14.6%) have been reported for spruce and pine plantations in Canada, particularly in cases where leaf-off imagery has been used in the analysis [38,69]. The commission errors in the present study could possibly be further reduced by applying more advanced methods for feature optimization in the creation of the tree cover mask, for example using classification trees [71]. Alternatively, a canopy height model derived from airborne LiDAR or alternatively HSR satellite stereo imagery [72] could be used to mask out small trees below a certain height and thus minimize commission errors resulting from tree re-growth.

### 4.2. Delineation Accuracy

**Figure 6.**
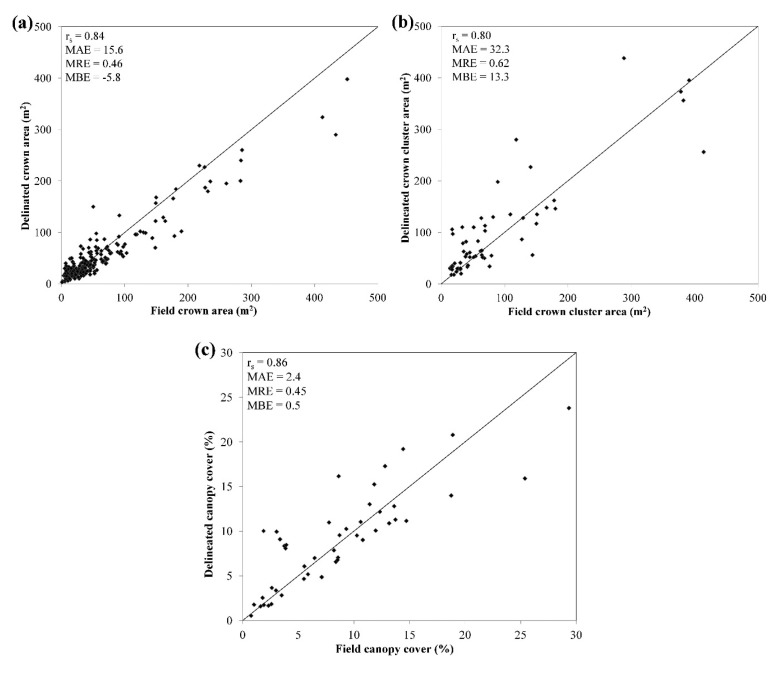
Graphs of the correlation between field data and remote sensing data (delineated) showing (**a**) crown area of ITC; (**b**) crown cluster area; and (**c**) tree canopy cover.

Results from the delineation accuracy assessment are presented (Table 8 and Figure 6) for different levels of tree crown aggregation (ITC and crown clusters), tree crown size classes (small, medium and large) and land-use categories (active field and fallow). The correlation coefficients (*r_s_*) presented in Table 8 and Figure 6 are all highly significant (<0.05) and show that the delineation accuracy is a function of tree crown size with lowest *r_s_* for small trees (0.419) and highest *r_s_* for large trees (0.905). The MRE shows a similar pattern: the delineation of small tree crowns results in the highest relative error (65%) and the delineation of large tree crowns results in the lowest relative error (21.3%). Furthermore, assessment of the individual errors revealed that the delineation tended to overestimate the CA of small trees (MBE = 4.0), whereas it underestimated the CA of medium and large trees (MBE = −41.8). Overestimation of CA for small trees was primarily caused by the presence of adjacent field layer vegetation that diluted the crown edges in the NDVI image used for region-growing segmentation. Furthermore, small trees cause less distinct shadows which reduced the effectiveness of using NIR reflectance for crowns edge detection in HSR imagery. The underestimation of large trees was primarily the result of high spectral variance near crown edges where the foliage was more open and where field layer reflectance had a strong influence on the spectral response. Such within-crown spectral variance was in some cases confused for crown edges by the region-growing segmentation. This effect was especially apparent for tree species characterized by expansive branching (e.g., *Parkia bigloba* and *Adansonia digitata)*. Thus, our results from using NDVI and NIR, in combination with thresholds optimized for tree species types (Section 3.1.2) in the region-growing segmentation indicates two things. Firstly, the approach was moderately successful in delineating small tree crowns, in particular those which display low contrast to the field layer. Secondly, the higher delineation accuracies for medium and large trees suggest that the approach reduces the effect of within-crown spectral variance, but does not remove it completely.

The overall delineation accuracy of ITC in this study (*r_s_* = 0.836, MAE = 15.6 m^2^, MRE = 45.6%) is in line with previous research. For example, Ardila *et al.*, reported a relative error of 40% compared to a manually delineated reference dataset [48]. Brandtberg and Walter reported a relative error of 46% when delineated ITC were compared to a field reference dataset [73]. Delineation errors as low as 17.9% have been achieved in even aged and well-spaced plantation forests [69]. However, lower delineation accuracies are to be expected in situations where the tree cover is characterized by high heterogeneity in terms of tree crown size distribution [20] and tree species diversity [34]. Consequently, the delineation accuracy achieved here must been contrasted to the wide tree crown size range (CA = 1–600 m^2^) and the relatively high tree species diversity observed in the study area.

The delineation accuracy for crown clusters is lower than for ITC, expressed in *r_s_* (0.800), MAE (32.3 m^2^) and MRE (61.5%). To derive “field crown cluster area”, the CA of individual trees measured in the field and included in the delineated crown cluster was aggregated without taking into account potential crown overlaps (Section 2.3). Since large trees in the clusters are likely to overtop, and thus obscure others from the view of the satellite sensor, this approach tends to overestimate field crown cluster area. An accurate delineation is therefore expected to underestimate crown cluster area to some degree. This effect is discernible in the active field plots (Table 8), but not in the fallow plots where overestimation of crown cluster area occurred due to the erroneous inclusion of adjacent field layer vegetation.

The tree canopy cover derived from the delineation agrees well with the reference data (Table 9; Figure 6c) in terms of *r_s_* (0.859) MAE (2.4%) and MRE (45%). The agreement is considerably better for plots in active fields (*r_s_* = 0.965) compared to fallow (*r_s_* = 0.707). Since the reference tree canopy cover was calculated by aggregating the CA of all trees on the plot level, some overestimation is induced because large trees may overtop or overlap (smaller) adjacent trees. This means that the delineation is expected to underestimate tree canopy cover slightly, which is the case for the plots in active fields (MBE = −1). However, since every delineated object (including commission errors) is used to calculate tree canopy cover, the underestimation is expected to be reduced. This is especially apparent in the fallow plots where the delineation overestimates tree canopy cover as a result of the higher frequency of commission errors (Figure 5b). Our results compare well with similar studies in terms of accuracy. For example, Rasmussen *et al.*, reported an agreement of *r*^2^ = 0.51 between observed (field) and delineated tree canopy cover for an open woodland area in northern Senegal [21], whereas Morales *et al.*, reported *r*^2^ = 0.86 and MAE = 1.9% in a Hawaiian dry forest [74].

In general, the delineation accuracies for ITC, crown clusters and tree canopy cover were consistently lower in the fallows compared to the active fields. Two main factors cause this difference. Firstly, the higher abundance of field layer vegetation in the fallows complicated the crown edge detection in region-growing segmentation. Secondly, the trees in fallows were generally smaller (mean CA = 51.4 m^2^) than trees in active fields (mean CA = 66.0 m^2^) and therefore more difficult to delineate (Table 8). This difference in tree size distribution is likely the results of local land management where a high proportion of the small trees are removed, while large trees are retained when the land is cleared prior to cultivation [6].

### 4.3. Considerations for Wider Application

The tree crown mapping approach presented in this article was designed to account for variability in tree crown size, canopy spectral properties (e.g., different tree species) and field layer conditions. The design included use of geometric and spectral thresholds for the separation of tree cover from field layer components (3.1.1. and 3.1.6.), the definition of crown edges (3.1.2. and 3.1.4.) and the classification of ITC and crowns clusters (3.1.3 and 3.1.6.). These thresholds were determined from a limited, yet structurally complex, 10 km × 10 km study area. It is reasonable to believe that the geometric thresholds will remain consistent for other woodland areas with similar vegetation characteristics, even in cases when imagery from different HSR remote sensing systems or vegetation seasons is used. However, attention should be given to the sun-sensor geometry at the time of image acquisition since this may significantly affect the visibility and appearance of trees in HSR imagery [41], especially for tree cover with mixed tree sizes and heights [20]. The spectral thresholds, on the other hand, are to some extent sensor (WorldView-2) specific, in particular those that are based on red edge reflectance. The specific values of the single band thresholds (e.g., NIR and red edge) applied in this study would need to be adjusted to account for differences in vegetation and atmospheric conditions. Identification of optimal spectral threshold values should be based on empirical evaluation, a process that is significantly aided by a priori knowledge about the structure and spectral properties of the local tree species and field layer components. Given the massive amount of potential object features available in GEOBIA [51], improvements in detection and delineation accuracies may be achieved by application of advanced optimization procedures for feature selection and threshold determination e.g., [71].

Similar to related research, we found that detection and delineation accuracies are higher in areas were the tree density is low and the understory vegetation is largely senescent. This suggests that the proposed combination of GEOBIA and HSR imagery is most useful for tree crown mapping in sparse woodlands, such as the West African parklands, whereas further research will be required to ensure accurate application in denser woodlands. Our results further suggest that in cases where trees crowns are strongly interlocked and arranged in compact crown clusters, individual tree crown mapping using HSR imagery may not always be a plausible expectation. Under these conditions, ITC delineation based on spectral gradients (e.g., NDVI and NIR) and object shape characteristics *(i.e.*, watershed segmentation) may be less successful. However, previous research argues that the mapping of crown clusters provides a useful means to model and analyze structural tree cover attributes, especially in areas characterized by high spatial heterogeneity such as African woodlands [41,54,55,75].

Preliminary results based on the reference dataset show strong relationships between delineated CA and tree height (*r_s_* = 0.711) and DBH (*r_s_* = 0.735), which suggests that the proposed method can be used to estimate these and related structural variables (e.g., biomass) over relatively large areas. Further analysis will be required to establish relationships between cluster area and tree cover structure in parklands and other woodland areas. Moreover, accurate maps of crown clusters and associated spatial attributes (e.g., size and shape) are highly relevant for ecological research targeting woodland and savanna landscapes in which the concept of patch dynamics is central [75,76]. The approach presented in this article represents an efficient means to extract detailed tree cover data from easily accessible, albeit relatively expensive, satellite imagery. Such spatially explicit datasets are critical for inventory and monitoring of African woodlands and facilitate improved understanding of tree cover structure, composition and dynamics. Detailed datasets of tree cover structure also provides a practical means to scale field observation to medium and coarse spatial resolution RS data, thereby facilitating monitoring at multiple spatial scales e.g., [77].

## 5. Conclusions and Future Research

This study aimed to evaluate the effectiveness of using WorldView-2 satellite imagery and GEOBIA for automating tree crown mapping in African woodlands, in particular agroforestry parkland landscapes. The geographical focus is one of the main contributions of this work since limited research has targeted the potential of HSR satellite imagery for forestry applications in African woodlands. HSR satellite systems represent a feasible alternative to airborne RS systems, especially in Africa where the availability of such equipment is highly limited.

The results show that reasonably accurate tree crown maps can be extracted from HSR satellite imagery (WorldView-2) by using a combination of spectral and geometric information derived from GEOBIA. The moderate detection accuracies (48.4%–53.8% for ITC) agree with previous research on tree crown mapping in complex environments and reflect the difficulty of resolving individual tree crowns in HSR imagery, especially in cases where tree crowns are interlocked to form compact crown clusters. The results improved considerably (85.7% accuracy for ITC and crown clusters) when crown clusters were considered as correct detections, suggesting this to be a reasonable mapping approach given constraints imposed by imagery characteristics and tree cover conditions. In a later stage of the analysis, ITC and crown clusters can be differentiated using the geometric information (e.g., shape and size) inherent to GEOBIA. The results further show that ITC and crown cluster area can be estimated with reasonable accuracy from the delineated objects. For small trees (<35 m^2^), delineation accuracy is relatively low (*r_s_* = 0.419; MRE = 65%) and the crown area is overestimated. For medium (35–100 m^2^) and large (≥100 m^2^) trees, the delineation accuracy is considerably higher (*r_s_* = 0.836–0.905; MRE = 21.3%–26.8%) and crown area is slightly underestimated. Delineation accuracy for crown clusters is lower (*r_s_* = 0.800; MRE = 61.5%), in part possibly reflecting the complicated task of measuring crown cluster area in the field.

Detection and delineation accuracies are consistently lower in the fallows compared to active fields for three reasons: (i) tree density is higher; (ii) trees size is smaller and (iii) the understory vegetation is denser. The dense understory vegetation in fallows is to a large extent composed of trees with DBH < 5 cm (e.g., re-growth), which causes high commission error rates. Substantial improvements in terms of commission error, as well as delineation accuracy, may be achieved by developing the method used for extracting the tree cover mask (Section 3.1.1). For example, future research should investigate the potential of advanced feature optimization procedures which may make better use of the vast spectral, geometric, textural and relational information available in HSR imagery through the application of GEOBIA. Alternatively, use of 3D information such as that derived from airborne LiDAR or HSR satellite imagery acquired in stereo pairs provides a means to derive canopy height models. Such models may both enable a more effective exclusion of understory vegetation and improve the possibilities to detect tree crown edges through region-growing segmentation.

The results of this work were limited to the 100 km^2^ study area located in the parklands of Saponé, Burkina Faso. Similar results, in terms of detection and delineation accuracies, are expected when the proposed method is applied in areas with similar tree cover structure. However, adjustment of thresholds, in particular those based on spectral information, may be required in order to account for differences in atmospheric and tree canopy conditions, and imagery characteristics. For following research, the application of the GEOBIA approach in other woodland and parkland areas is necessary to assess its consistency and transferability.

## Figures and Tables

**Table 1.** Technical specifications of the WorldView-2 image acquired 21 October 2012 (Source: Digital Globe 2013a).

**Band**	**Wavelength Region (μm)**	**Pixel Size (m)**		
Panchromatic	0.450–0.800	0.5	Acquisition date	21 October 2012
Coastal blue	0.400–0.450	2	Acquisition time	13:55:15
Blue	0.450–0.510	2	Mean off-nadir angle	12.9°
Green	0.510–0.580	2	Mean sun azimuth	153.4°
Yellow	0.585–0.625	2	Mean sun elevation	64.5°
Red	0.630–0.690	2	Mean satellite azimuth	192.4°
Red edge	0.705–0.745	2	Mean satellite elevation	75.3°
NIR 1	0.770–0.895	2		
NIR 2	0.860–1.040	2		

**Table 2.** Characteristics of the stratification used during field work, including tree canopy cover (CC), tree density (TD), standard deviation (s.d.) and distribution of field plots.

**Strata**	**CC (%)**	**TD^ha − 1^ Min**	**TD^ha − 1^ Max**	**TD^ha − 1^ Average**	**TD^ha − 1^ s.d.**	**Plots Total**	**Plots in Active Field**	**Plots in Fallow**
1	0	-	-	-	-	-	-	-
2	≥0–10	4	56	21	14	24	16	8
3	≥10–40	8	64	32.5	14.6	23	10	13
4	≥40	24	208	96.4	54.4	18	4	14
Total		4	208	46.6	44.5	64	29	35

**Table 3.** Data collected for each tree during fieldwork.

**Surveyed Variables**	**Comment**
Tree ID	Unique identifier
Location	X,Y using GPS (Garmin Oregon 550)
Species	Identified by local botanist
DBH (cm)	Tree stem diameter measured at 1.3 m above ground
Tree height (m)	Haglof Electronic Clinometer
Crown diameter (D1; m)	Below crown distance measurements of the largest axis using tape measure
Crown diameter (D2; m)	Below crown distance measurements of the axis perpendicular to D1

**Table 4.** Object features used for tree mask generation and tree crown identification.

**Object Feature**	**Threshold**	**Equation**	**Values**
Area (m^2^)	<1500 ^◊^	a = n × p	a = area
			*n* = number of pixels
			p = pixel size (m^2^)
Roundness	<0.6 ^◊^	r = s − l	r = roundness
			s = radius of smallest enclosing ellipse
			l = radius of largest enclosing ellipse
Elliptical fit *	<0.7 ^◊^		
NIR (s.d.)	>8 ^◊^ <4 ^□^	$\sigma =\sqrt{\frac{1}{n}\times \overset{n}{\underset{i=1}{\text{∑}}}{\left(C_{i}-C\right)}^{2}}$	*σ* = s.d.
			C = mean NIR
			C_i_ = NIR values
			*n* = number of pixels in object
Red edge (s.d.)	<3 ^□^	$\sigma =\sqrt{\frac{1}{n}\times \overset{n}{\underset{i=1}{\text{∑}}}{\left(D_{i}-D\right)}^{2}}$	*σ* = s.d.
			D = mean RE
			D_i_ = RE values
			*n* = number of pixels in object

* Equation can be found in [65];

◊ Used for iterative NDVI threshold in tree cover mask generation;

□ Used for multi-resolution segmentation in tree cover mask generation and for individual tree crown identification (Section 3.1.6).

**Table 5.** Classification of seeds to tree species type and region-growing thresholds established from the training dataset.

**Tree Species Type**	**Mean NDVI**	**NDVI Threshold**	**NIR Threshold**
Species 1	0.1–0.2	0.08	30
Species 2	>0.2–0.3	0.15	40
Species 3	>0.3	0.18	50

**Table 6.** Definitions of measures used for detection accuracy assessment.

**Measure**	**Description**	**Unit**
ITD	Individual tree detection	%
CCD	Crown cluster detection	%
OE	Omission error	%
CE	Commission error	%

**Table 7.** Results of detection accuracy assessment showing detection rate (DR; %), omission error (OE; %), commission error (CE; %) and accuracy index (AI; %).

**Land Use**	**Field Trees**	**DR (ITC)**	**DR (ITC and Crown Clusters)**	**OE**	**CE**	**AI**
Active field	189	55 ^1^/60.7 ^2^	88.4 ^1^	11.6 ^1^/5.3 ^2^	12.7	76 ^1^/79 ^2^
Fallow	308	43.8 ^1^/48.4 ^2^	87.7 ^1^	12.3 ^1^/9.3 ^2^	21.7	66 ^1^/64.5 ^2^
Total	497	48.4 ^1^/53.8 ^2^	85.7 ^1^	14.3 ^1^/6.6 ^2^	18.3	67.4 ^1^/69.7 ^2^

1 Trees with dbh > 5 cm;

2 Trees with dbh > 10 cm.

**Table 8.** Results of accuracy assessment for the delineation of ITC and crown clusters presented by Spearman's Rho correlation coefficient (*r_s_*), mean absolute error (MAE), mean relative error (MRE), and mean bias error (MBE).

**Level of Aggregation**	**Category**	***r****_s_*	**MAE (m^2^)**	**MRE (%)**	**MBE (m^2^)**	**n**
ITC	Small CA (<35 m^2^)	0.419	8.5	65.0	4.0	122
	Medium CA (35–100 m^2^)	0.613	15.2	26.8	−6.9	85
	Large CA (≥100 m^2^)	0.905	44.5	21.3	−41.8	31
	All (active field)	0.877	16.5	30.0	−12.5	107
	All (fallow)	0.804	14.8	58.0	−0.5	131
	All (fallow + active field)	0.836	15.6	45.6	−5.8	238
Crown clusters	Active field	0.860	27.2	32.7	−7.1	21
	Fallow	0.739	35.0	77.0	24.3	39
	All	0.800	32.3	61.5	13.3	60
ITC and crown clusters	Active field	0.892	18.2	30.7	−11.7	128
	Fallow	0.808	19.5	62.5	5.2	170
	All	0.836	18.9	48.8	−2.0	298

**Table 9.** Results of accuracy assessment for the delineation of tree canopy cover presented by Spearman's Rho correlation coefficient (*r_s_*), mean absolute error (MAE), mean relative error (MRE), and mean bias error (MBE). Ease.

**Category**	***r****_s_*	**MAE (%)**	**MRE (%)**	**MBE (%)**	**n**
Active field	0.965	2.1	24	−1	22
Fallow	0.707	2.7	64	1.8	24
All	0.859	2.4	45	0.5	46
